# Differential potentiation of odor aversion and yawning by melanocortin 4 receptor signaling in distinct regions of the ventral striatum

**DOI:** 10.3389/fnins.2025.1668410

**Published:** 2025-11-04

**Authors:** Md Tasnim Alam, Md Monjurul Ahasan, Shogo Shimizu, Yoshihiro Murata, Mutsuo Taniguchi, Masahiro Yamaguchi

**Affiliations:** Department of Physiology, Kochi Medical School, Kochi University, Kochi, Japan

**Keywords:** olfactory tubercle, nucleus accumbens, neuromodulatory signal, melanocortin 4 receptor, olfactory behavior, yawning

## Abstract

Although animal behavior is influenced by neuromodulatory signals, the underlying mechanism remains elusive. The ventral striatum, which consists of the olfactory tubercle (OT) and nucleus accumbens (NAc), promotes motivated behaviors and receives substantial neuromodulatory signals. We previously showed that the OT has anteromedial (am) and lateral domains regulating odor-guided attractive and aversive behaviors, respectively, in which the amOT highly expresses various receptors for feeding-regulated neuromodulators. Here, we investigated the functions of appetite-suppressing melanocortin 4 receptor (MC4R) signaling in the OT as well as in the NAc. When mice conditioned with an odor-food reward association underwent MC4R agonist injection in the amOT, their odor-attractive behavior was suppressed and odor-aversive behavior was induced. Conversely, injection of MC4R antagonist in the amOT induced attractive behavior to a neutral odor that was not associated with food reward. While MC4R agonist injection in the NAc shell did not influence odor-attractive behavior, it induced yawning and stretching behaviors. Consistent with a proposed role of these behaviors in the thermoregulation of the brain, recordings of brain temperature showed its occasional elevation after agonist injection, followed by the occurrence of yawning and stretching. These observations demonstrate the differential roles of MC4R signaling in the ventral striatum, the promotion of odor-aversive behavior in the amOT, and yawning/stretching behavior in the NAc, which are considered to collectively contribute to behavioral control under feeding.

## Introduction

Molecules and neural circuits involved in energy balance also play a significant role in regulating affective states (Liu et al., 2014). The interplay between circuits governing energy homeostasis and those involved in reward and motivation has proven essential for understanding feeding behavior (Rolls, 2016; Waterson and Horvath, 2015). The ventral striatum is a key region in this process, orchestrating affective and motivated behaviors through extensive dopaminergic input from the ventral tegmental area of the midbrain (Ikemoto, 2007). Notably, the ventral striatum integrates energy-related signals from both central and peripheral systems, including the hypothalamus, and modulates motivated behaviors in accordance with the organism’s energy status (Ferrario et al., 2016; Jin et al., 2023).

The ventral striatum consists of the nucleus accumbens (NAc) and the olfactory tubercle (OT) (Ikemoto, 2007). The OT, also referred to as the tubular striatum (Wesson, 2020), is part of the olfactory cortex and receives direct synaptic input from the olfactory bulb (Neville and Haberly, 2004; Wesson and Wilson, 2011). It encodes odor valence and contributes to a range of odor-guided behaviors (DiBenedictis et al., 2015; Fitzgerald et al., 2014; Gadziola et al., 2015). We previously showed that the OT in mice has anteromedial (am) and lateral (l) domains, which regulate odor-guided attractive and aversive behaviors, respectively (Murata et al., 2015). Notably, the amOT exhibits high expression of receptors for feeding-regulated neuromodulators (Nogi et al., 2020), suggesting that it plays a key role in modulating odor-guided motivational behaviors in accordance with energy and metabolic status. Indeed, it expresses the appetite-promoting orexin 1 receptor (OxR1) at high levels, and local injection of an OxR1 antagonist into the amOT has been shown to shift the valence of a food reward-associated odor from attraction to aversion (Ahasan et al., 2024). Moreover, orexin enhances synaptic plasticity within the amOT (Podder et al., 2024). Collectively, these findings support the notion that the amOT integrates energy/metabolic signals to regulate odor valence.

The amOT also expresses the appetite-suppressing melanocortin type 4 receptor (MC4R) at high levels (Nogi et al., 2020). MC4R is activated by *α*-melanocyte stimulating hormone (α-MSH), which is primarily produced by pro-opiomelanocortin (POMC) neurons in the arcuate nucleus of the hypothalamus (Krashes et al., 2016). The melanocortin–MC4R system is a central regulator of feeding behavior and energy expenditure (Baldini and Phelan, 2019; Krashes et al., 2016). MC4R is widely expressed across brain regions beyond the hypothalamus, with particularly high expression in the NAc (Kishi et al., 2003; Siljee-Wong, 2011). Within the NAc, MC4R activity suppresses both appetitive and consummatory behaviors (Eliason et al., 2022; Lerma-Cabrera et al., 2012). Moreover, melanocortin–MC4R signaling in the NAc is essential for non-appetitive aversive behaviors, including anhedonia and escape responses to aversive stimuli (Klawonn et al., 2018; Lim et al., 2012). These findings underscore the critical role of MC4R signaling in the NAc in regulating a broad spectrum of motivated behaviors.

While MC4R expression is high in the OT among olfactory cortical regions (Kishi et al., 2003), the functional role of the melanocortin–MC4R system in the OT remains poorly understood. In this study, we examined the role of MC4R in the OT by locally injecting a receptor agonist and antagonist, and identified the amOT as a key region involved in odor valence regulation.

During this analysis, we unexpectedly observed that MC4R agonist injection into the NAc promoted yawning and stretching behaviors. Although the physiological significance of yawning and stretching remains debated (Gupta and Mittal, 2013; Krestel et al., 2018), several studies have proposed a role in brain thermoregulation (Gallup and Eldakar, 2012; Shoup-Knox et al., 2010). Consistent with this hypothesis, we measured brain temperature before, during, and after MC4R agonist injection in the NAc and found that temperature elevation preceded the onset of yawning and stretching.

## Materials and methods

### Animals

All experimental procedures were conducted according to the guidelines of the Physiological Society of Japan and were approved by the Animal Care and Use Committee of Kochi Medical School. Male C57BL/6 mice (Japan SLC Inc., Shizuoka, Japan) were housed individually in a plastic cage (24 × 17 × 12 cm) with wood shavings under a controlled temperature of 26 °C and 12 h light /dark cycle (lights were switched on at 21:00; off at 09:00).

### Implantation of guide cannulas for drug injection and brain temperature recording

Male mice (8 weeks old) were used for stereotaxic surgery. The mice were anesthetized intraperitoneally with a mixture of three anesthetics (0.3 mg/kg medetomidine, 4 mg/kg midazolam, and 5 mg/kg butorphanol) and placed on a stereotaxic frame. Then, the shaved skull was exposed through a midline incision, scratched, and cleaned to remove the periosteum. The skull was drilled to make an appropriate hole at the position of cannula implantation. For bilateral implantation in the amOT, to avoid damage to midline vessels, the bone directly over the midline was left intact. Stainless steel 26-gage guide cannulas (1.8 cm in length) with 31-gage obturators, which were designed to extend 1 mm beyond the guide cannulas, were implanted into the bilateral amOT (2.0 mm anterior to the bregma, 0.3 mm lateral to the midline, 4.0 mm deep from the brain surface), lOT (1.9 mm anterior to the bregma, 1.4 mm lateral to the midline, 4.0 mm deep), or NAc shell (2.0 mm anterior to the bregma, 0.7 mm lateral, 3.2 mm deep). The cannula in the NAc shell was placed at its anteromedial region, dorsal to the amOT. Cannulas were fixed to the skull using dental cement, which was allowed to fully harden before removing the cannula holder. The implants were protected by a plastic cup fashioned from a 15-mL plastic tube. The stereotaxic coordinates of the cannula location described above indicated the position of the tip of the obturator.

For brain temperature recording, a 26-gage guide cannula (1.8 cm in length) with a 31-gage obturator was implanted unilaterally in the lateral NAc (2.0 mm anterior to the bregma, 1.4 mm lateral, 3.2 mm deep). Guide cannulas for MC4R agonist or vehicle injection were implanted bilaterally into either the NAc shell or amOT, as described above.

In total, 52 mice were used for all analyses in the present study. Number of mice used in each experiment was described in corresponding figure legends.

### MC4R agonist/antagonist preparation and injection

MC4R agonist [cyclo(*Β*-Ala-His-D-Phe-Arg-Trp-Glu)-NH_2_] (Phoenix Pharmaceuticals Inc., Burlingame, CA, USA) was dissolved in 0.9% sodium chloride at a concentration of 1.87 μg/μL. Mice were gently held by hand, and the obturator of the drug cannula was removed. Then, a 31-gage inner cannula was inserted into the target brain region, which was connected to a syringe pump (SP101i, World Precision Instruments Inc., Sarasota, FL, USA) with polyethylene tubes (SP10 and SP19, Natsume Seisakusho Co., Ltd., Tokyo, Japan). The inner cannula was designed to extend 1 mm beyond the guide cannula. For each side, the drug solution was injected at a rate of 0.08 μL/min for 5 min (0.4 μL total volume; 0.75 μg MC4R agonist). Following injection, the cannula was held in place for an additional 5 min (total handling time ~15 min), after which the mice were returned to their home cages. For vehicle injections, a 0.9% sodium chloride solution was used.

For MC4R antagonist delivery, HS014 [Ac-Cys-Glu-His-{D-2Nal}-Arg-Trp-Gly-Cys-Pro-Pro-Lys-Asp-NH_2_] (Phoenix Pharmaceuticals) was dissolved in 0.9% sodium chloride at a concentration of 0.1 μg/μL. The antagonist solution was injected using the same parameters, 0.08 μL/min for 5 min (0.4 μL total volume; 0.04 μg MC4R antagonist per side).

### Odor–sugar association training

Odor-sugar association training was conducted as reported previously (Ahasan et al., 2024) with minor modifications. Association training began after a 2-week recovery period. During training, mice were food-restricted to 80–90% of their *ad libitum* body weight by providing a limited amount of food (2.7–3.3 g/day). Food restriction commenced 2 days before the onset of odor–sugar association training. Water was available *ad libitum* throughout the experiment.

Each mouse was trained individually in a plastic conditioning cage (24 × 17 × 12 cm) filled with 2 cm of white paper bedding (Japan SLC Inc.). On the first day of training, mice were habituated to sugar by being presented with granulated sucrose (20–40 mg) alongside powdered diet (20–40 mg) on a Petri dish, followed by sugar alone (50 mg).

Subsequently, mice underwent odor–reward association training using eugenol as the cue odor. Sugar (50 mg) was placed on a holed Petri dish, beneath which a 2 × 2 cm piece of filter paper soaked with 10 μL of eugenol (Tokyo Chemical Industry, Tokyo, Japan) was affixed to the bottom section. Mice were allowed to consume the sugar while detecting the eugenol odor; this procedure was repeated three times. On training days 2–4, the dish was buried 2 cm beneath the bedding, and its position within the cage was randomized to require odor-guided search. Because mice reliably located and consumed the sugar within 2 min, each trial was limited to a 2-min duration. Four training trials were conducted per day.

For mice that did not undergo odor–sugar association training, the same food restriction protocol was used. These mice received a holed Petri dish containing eugenol-soaked filter paper (10 μL), but no sugar, for 2-min trials. Four trials were conducted per day across training days 1–4.

### MC4R agonist/antagonist injection and behavior analysis

Following 4 days of training on odor–sugar associations, the effect of MC4R agonist injection on odor-guided behavior was assessed using a between-subjects design. On test day 1, mice were habituated to a larger test cage (30 × 20 × 13 cm) containing a 2-cm-deep layer of paper bedding for 30 min. Then, they were divided into two groups that received injections of MC4R agonist or vehicle into the bilateral amOT, lOT, or NAc shell via drug cannulas. After a 30-min rest in their home cages, mice were placed into the test cages, where dishes scented with 10 μL of eugenol but lacking sugar were buried under the bedding. Digging behavior was video-recorded for 2 min and analyzed offline. Digging at the odor location was defined as digging over the buried odor-containing dish, and digging outside the odor location was defined as digging elsewhere in the cage. Total digging time during the 2-min trial was calculated. Each mouse underwent four trials with 1-min intervals. Total digging time across the four trials was quantified and analyzed. This procedure was repeated on test days 2 and 3.

On test day 4, the mice were habituated to a bedding-free test cage (30 × 20 × 13 cm), and then injected with MC4R agonist or vehicle via the same cannulas. After a 30-min rest in the home cages, the mice were transferred to the test cages, where dishes scented with 10 μL of eugenol (without sugar) were placed openly. Behavior was video-recorded for 2 min and analyzed offline. Investigating the odor was defined as nose positioning within 1 cm of the dish with focused attention. Retraction behavior was defined as an initial approach to the dish followed by a rapid withdrawal (whole-body flinch). During behavioral analysis, the cage space was divided at the midline into two zones: the odor-containing zone (zone 1) and the opposite side (zone 2). The time spent in each zone was estimated using SMART ver. 3.0 software (Panlab, Barcelona, Spain). Each mouse underwent four trials with 1-min intervals. Total behavioral times across the four trials was quantified and analyzed.

Following the day 4 test, mice were given sufficient food. On test day 5, after ~24 h of *ad libitum* feeding, mice underwent the same procedures as on test day 4: cage habituation, MC4R agonist or vehicle injection, and behavioral testing in response to odor presentation.

To assess the effect of MC4R antagonist injection on an odor with neutral valence, the same odor (eugenol) was presented to food-restricted mice without sugar for 4 days. Then, the behavior in response to odor presentation was evaluated following MC4R antagonist or vehicle injection into the amOT using the same procedures as in the MC4R agonist experiments.

**Figure 4:**
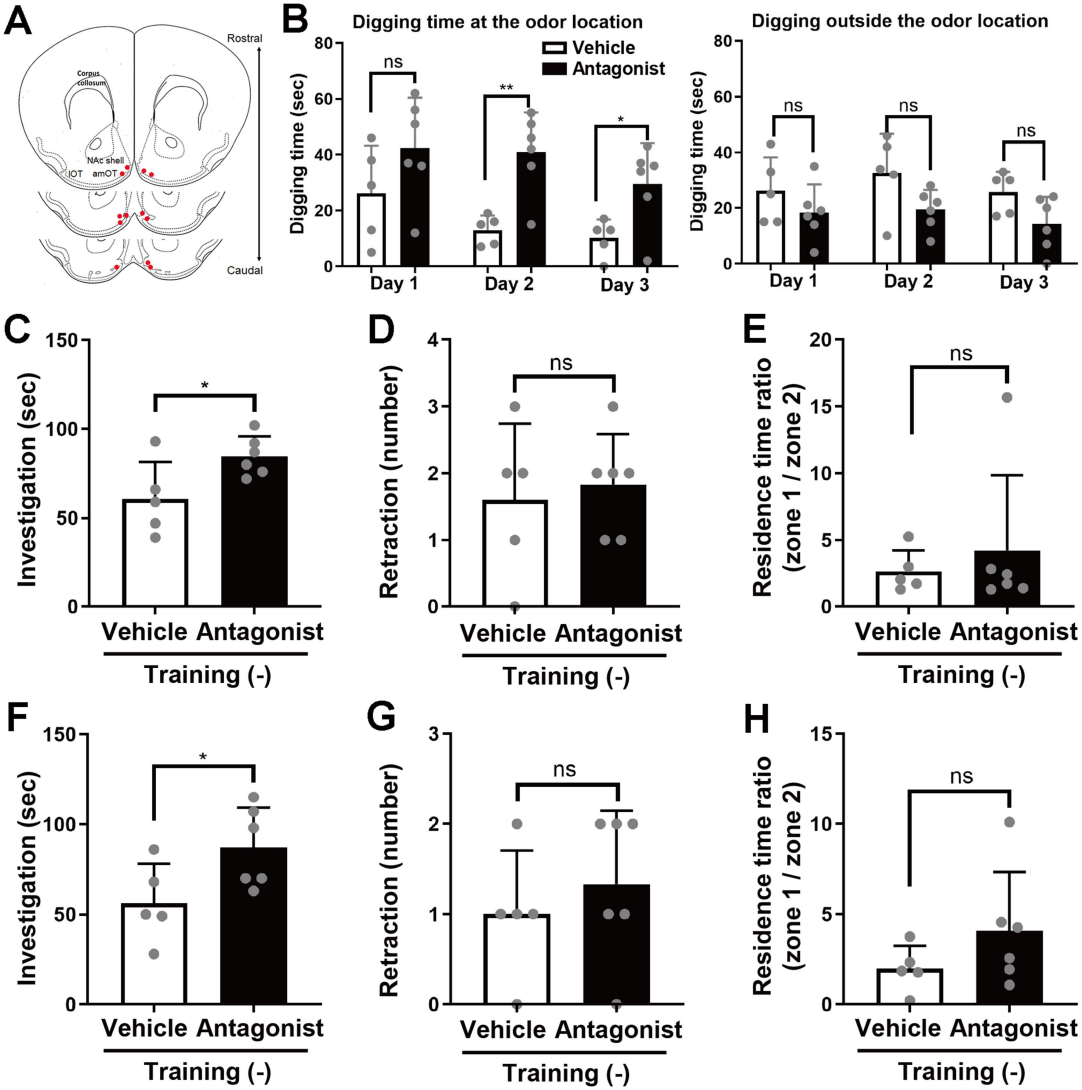
Conversion of the unassociated odor valence from neutral to aversion by local injection of an MC4R antagonist into the amOT. **(A)** Schematic of cannula placement (red dots; *n* = 6 antagonist-injected mice). **(B)** Odor-guided behavior of mice that did not receive odor–sugar association training, during test days 1–3. (Left) Digging behavior at the odor location. (Right) Digging behavior outside the odor location. Digging time within four 2-min trials of vehicle-injected mice (white columns) and antagonist-injected mice (black columns). **(C–E)** Odor-guided behavior of food-restricted mice under the bedding-free condition. **(C)** Odor-investigating time within four 2-min trials of vehicle-injected mice (white column) and antagonist-injected mice (black column). **(D)** Occurrence of odor-retraction within four 2-min trials of two groups of mice. **(E)** Ratio of residence times on the odor side (zone 1) versus the opposite side (zone 2) of two groups of mice. **(F–H)** Odor-guided behavior of *ad libitum*-fed mice under the bedding-free condition. **(F)** Odor-investigating time within four 2-min trials of vehicle-injected mice (white column) and antagonist-injected mice (black column). **(G)** Occurrence of odor-retraction within four 2-min trials of two groups of mice. **(H)** Ratio of residence times on the odor side (zone 1) versus the opposite side (zone 2) of two groups of mice. Average ± standard deviation, each dot represents one mouse (*n* = 5 mice for vehicle-injected group; *n* = 6 for antagonist-injected group). ns, not significant; *, *p* < 0.05; **, *p* < 0.01 (*t*-test).

### Brain temperature recording and yawning/stretching behavior analysis

Mice were subjected to temperature recordings after a 2-week recovery period following implantation of guide cannulas. During this period, mice were habituated to restraint in a hollow plastic tube to ensure immobilization during bilateral drug injections, as well as to freely moving conditions in a plastic cage (24 × 17 × 12 cm) following release from the tube.

For temperature recording, a thermocouple probe (PTW-300, Unique Medical Inc., Tokyo, Japan) was inserted through the guide cannula and extended 1 mm beyond its tip. The signal was digitized via a digital thermometer (PTW-400, Unique Medical) and processed using Spike II software (Cambridge Electronic Design Ltd., Cambridge, UK) with the sampling rate at 10 Hz. A total of 68 min of brain temperature was recorded. Mice were confined in hollow tubes for the initial 38 min (30 min pre-injection stabilization period and 8 min during drug or vehicle injection). MC4R agonist or vehicle (saline) was injected at a rate of 0.133 μL/min for 3 min (0.4 μL total volume) per side. Upon completion, mice were released from the tube, and temperature recording continued for an additional 30 min while they moved freely within the plastic cage (24 × 17 × 12 cm).

Mouse behavior during the free-moving period was recorded and analyzed for the yawning and stretching behavior. Yawning was defined when mice opened the mouth widely with the eyes closed and the behavior extended at least for several seconds. Stretching was defined when the mouse stretched the limbs with the trunk bending backward.

### Histological analysis

On the final day of behavioral analysis (day 5), mice were deeply anesthetized via intraperitoneal injection of sodium pentobarbital (150 mg/kg) at 1 h after the first odor presentation. Transcardial perfusion was performed using phosphate-buffered saline, followed by 4% paraformaldehyde in 0.1 M phosphate buffer. Brains were extracted, post-fixed in 4% paraformaldehyde overnight, and then cryoprotected in 30% sucrose in 0.1 M phosphate buffer. Subsequently, brains were embedded in optimal cutting temperature compound (Sakura Finetek, Tokyo, Japan), frozen at −80 °C, and sliced into 20-μm-thick coronal sections using a cryostat. Sections were mounted on glass slides.

To verify cannula placement, sections were stained with 4′,6-diamidino-2-phenylindole (DAPI; 2 μg/mL) and visualized under a fluorescence microscope. Mice with incorrect cannula placement were excluded from analysis. For brain temperature recordings, the location of the thermocouple guide cannula was also confirmed using the same procedure. Cannula placement was assessed based on visible tissue damage, which was primarily caused by the outer guide cannulas (26-gage). In contrast, the inner obturators and drug cannulas, designed with a thinner diameter (31-gage) and a fixed protrusion length of 1 mm from the guide cannula, produced minimal tissue disruption, even after repeated injections. In total, 27 mice were excluded due to incorrect cannula placement, many of which occurred during the early experimental period of the experimenter (Supplementary Figure 1). The exclusion criterion was based solely on cannula location. Following exclusion, the video-recorded behaviors of the mice with confirmed correct cannula placement were analyzed offline.

### Statistics

For the evaluation of digging behavior across days, two-way repeated measures of analysis of variance (ANOVA) (one repeated measures factor for “day” and one non-repeated measures factor for “injection”) was conducted using IBM SPSS Statistics software (ver. 23.0; IBM Corp., Armonk, NY, USA). For pairwise comparisons between vehicle- and agonist/antagonist-injected groups on each day, two-tailed Student’s *t*-tests were performed using Prism software (ver. 7.04; GraphPad Software, Inc., La Jolla, CA, USA). Statistical significance was defined as *p* < 0.05. Graphs were plotted using Prism software.

## Results

### Local injection of MC4R agonist into the amOT suppresses odor-attractive behavior and induces aberrant behavior toward the cue odor

**Figure 1:**
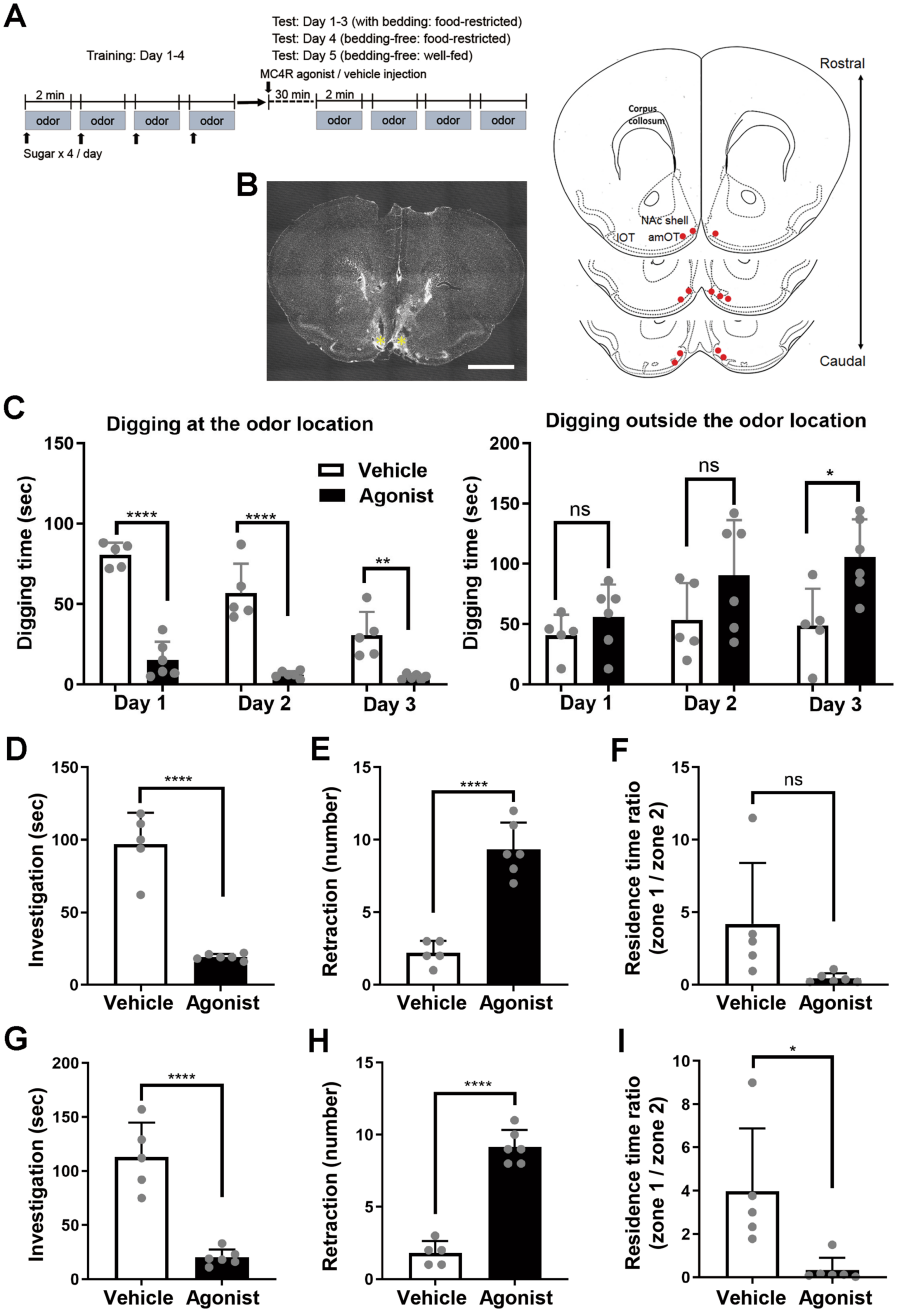
Suppression of odor-attractive behavior and promotion of odor-aversive behavior by local injection of an MC4R agonist into the amOT. **(A)** Protocol for odor–sugar association training and behavioral analysis following MC4R agonist injection. Food-restricted mice received association training with an odor (eugenol) and sugar for 4 days (training: day 1–4). The mice underwent injection of an MC4R agonist or vehicle in the bilateral amOT and were examined for odor-guided behavior for 5 days (test: day 1–5). Different experimental conditions among test day 1–3, 4 and 5 are indicated. **(B)** Placement of drug cannulas in the amOT. (Left) Coronal section of the OT. Asterisk, position of the cannula tip. Scale bar, 1 mm. (Right) Schematic of cannula placement (red dots; *n* = 6 agonist-injected mice). **(C)** Odor-guided behavior of mice during test days 1–3. (Left) Digging behavior at the odor location. (Right) Digging behavior outside the odor location. Digging time within four 2-min trials of vehicle-injected mice (white columns) and agonist-injected mice (black columns). **(D–F)** Odor-guided behavior of food-restricted mice under the bedding-free condition. **(D)** Odor-investigating time within four 2-min trials of vehicle-injected mice (white column) and agonist-injected mice (black column). **(E)** Occurrence of odor retraction within four 2-min trials in the two groups. **(F)** Ratio of residence time on the odor side (zone 1) versus the opposite side (zone 2) in the two groups. **(G–I)** Odor-guided behavior of *ad libitum*-fed mice under the bedding-free condition. **(G)** Odor-investigating time within four 2-min trials of vehicle-injected and agonist-injected mice. **(H)** Occurrence of odor retraction within four 2-min trials in the two groups. **(I)** Ratio of residence time on the odor side (zone 1) versus the opposite side (zone 2) in the two groups. In **C–I**, the average ± standard deviation are shown. Each dot represents one mouse (*n* = 5 for vehicle-injected group; *n* = 6 for agonist-injected group). ns, not significant; *, *p* < 0.05; **, *p* < 0.01; ****, *p* < 0.0001 (unpaired *t*-test).

Figure 1A shows the schedule of the behavioral analysis. Mice under food restriction received association training on a cue odor (eugenol) and sugar reward for 4 days. They were subsequently injected with an MC4R-specific agonist [cyclo(*Β*-Ala-His-D-Phe-Arg-Trp-Glu)-NH_2_] or vehicle (saline) via cannulas implanted in the bilateral amOT. Correct positioning of the cannulas was confirmed histologically after completion of the behavioral analyses (Figure 1B); data from mice with inappropriate cannula placement were omitted from the analysis.

On day 1 of the test period, 30 min after agonist or vehicle injection, mice were transferred to test cages where a eugenol-scented dish (without sugar) was buried under bedding on one side. Vehicle-injected mice extensively investigated the location of the cue odor (digging at the odor location) (Figure 1C, left panel; Supplementary Video 1). By contrast, agonist-injected mice spent significantly less time investigating the odor location (*F*(1, 9) = 129.0, *p* < 0.001 for injection; *F*(2, 18) = 10.68, *p* < 0.001 for interaction, two-way analysis of variance (ANOVA); *p* < 0.0001, *t*-test for day 1). The same procedure was repeated on test days 2 and 3. Over the 3-day test period, digging time at the odor location gradually decreased in both groups (*F*(2, 18) = 24.93, *p* < 0.001 for day, two-way ANOVA), consistent with extinction or re-learning of the odor–sugar association, because only the cue odor was presented without sugar. Even accounting for this decline in investigation, agonist injection continued to suppress odor-attractive behavior across all test days (test day 2, *p* < 0.0001; test day 3, *p* = 0.0018; *t*-test).

In addition, agonist-injected mice tended to dig the bedding outside the location of the cue odor (digging outside the odor location). Typically, they dug on the side opposite the odor source (*F*(1, 9) = 8.65, *p* = 0.016 for injection; *F*(2, 18) = 1.28, *p* = 0.30 for interaction, two-way ANOVA; Figure 1C, right panel; Supplementary Video 2). This aberrant behavior resembled that previously observed in mice that received OxR1 antagonist injection in the amOT (Ahasan et al., 2024). In the present study, the increase in digging outside the cue odor location was not significant on test day 1 or 2 (test day 1, *p* = 0.30; test day 2, *p* = 0.16; *t*-test), but became significant on test day 3 (*p* = 0.015; *t*-test).

Across the 3-day test period, the effect of MC4R agonist appeared to be potentiated. The percentages of digging time at the odor location for agonist-injected mice (average of six mice) versus vehicle-injected mice (average of five mice) were 19.0% (15.3 s/80.6 s) on day 1, 10.9% (6.2 s/56.8 s) on day 2, and 15.3% (4.7 s/30.6 s) on day 3. By contrast, the percentages of digging time outside the odor location were 137.7% (56.2 s/40.8 s) on day 1, 169.5% (90.5 s/53.4 s) on day 2, and 216.2% (105.5 s/48.8 s) on day 3. These findings indicate a progressive suppression of odor-attractive behavior and a corresponding potentiation of aberrant behavior (digging outside the odor location) over the 3-day test period.

Digging at the odor location is considered a food-searching behavior (Ahasan et al., 2024; Machado et al., 2018; Murata et al., 2015). By contrast, the digging behavior outside the odor location, potentiated in agonist-injected mice, may reflect a protective response to the cue odor, as it resembles burrowing behavior used to shelter from potential threats (Fucich and Morilak, 2018; Harper and Batzli, 1996), or it may reflect odor-induced anxiety that sometimes potentiates aberrant behaviors (Himanshu et al., 2020). These observations indicate that local injection of an MC4R agonist into the amOT suppresses odor-attractive behavior and suggest that this manipulation may conversely induce aversive responses to the cue odor.

### Local injection of an MC4R agonist into the amOT converts the odor valence from attraction to aversion

To evaluate whether agonist-injected mice developed aversion to the cue odor, their behavior was assessed in a bedding-free cage to eliminate confounding digging activity. On test day 4, mice were habituated to the bedding-free environment, injected with either agonist or vehicle into the amOT, and then exposed to the cue odor.

Vehicle-injected mice exhibited extensive investigative behavior toward the odor (Figure 1D; Supplementary Video 3). By contrast, agonist-injected mice spent significantly less time investigating the odor (*p* < 0.0001, *t*-test) and displayed retraction behavior (Figure 1E; Supplementary Video 4). Specifically, they approached the odor with their body in longitudinal extension, followed by a rapid withdrawal of the head and body (whole-body flinch). While the mice initially exhibited attention to the odor by orienting their noses toward it, approaching closely, and then retracting, they gradually began to display this attention-retraction behavior from increasingly distant locations relative to the odor source. This behavior was rarely observed in vehicle-injected mice (*p* < 0.0001, *t*-test) or in vehicle-injected control mice that had not undergone odor–sugar association training (see the data for untrained mice in the corresponding figure shown later). Although not statistically significant, agonist-injected mice also tended to spend less time in the odor-side zone (zone 1) compared to vehicle-injected mice (*p* = 0.055, *t*-test; Figure 1F). These findings indicate that MC4R agonist injection into the amOT suppresses odor-attractive behavior and induces aversive responses to the cue odor.

To determine whether this shift in odor valence was dependent on metabolic status, mice were provided *ad libitum* access to food following the day 4 test. After ~24 h of feeding, body weights returned to pre-restriction levels. The body weights of agonist-injected mice just before food restriction, during restriction (test day 4), and after *ad libitum* feeding (test day 5) were 25.7 ± 1.3 g, 22.2 ± 0.6 g (86.4 ± 2.4% of that just before food restriction), and 25.8 ± 1.5 g (100.3 ± 3.3%; *n* = 6), respectively. For vehicle-injected mice, the corresponding values were 25.7 ± 2.8 g, 22.0 ± 2.7 g (85.7 ± 4.1%), and 26.6 ± 2.6 g (103.8 ± 4.9%; *n* = 5). On test day 5, under the same bedding-free conditions, agonist-injected mice again showed reduced investigation of the cue odor (*p* < 0.0001, *t*-test; Figure 1G) and increased retraction behavior (*p* < 0.0001, *t*-test; Figure 1H), and spent less time in the odor-side zone (*p* = 0.014, *t*-test; Figure 1I) compared to vehicle-injected mice.

These results demonstrate that MC4R agonist injection into the amOT shifts odor valence from attraction to aversion, and that this effect persists regardless of metabolic status. Lack of the obvious effects of MC4R agonist injection in mice with inappropriate cannula placement supports involvement of the amOT in the odor valence control (Supplementary Figure 2).

### Local injection of an MC4R agonist into the lOT or NAc shell does not affect odor-guided attractive behavior

**Figure 2:**
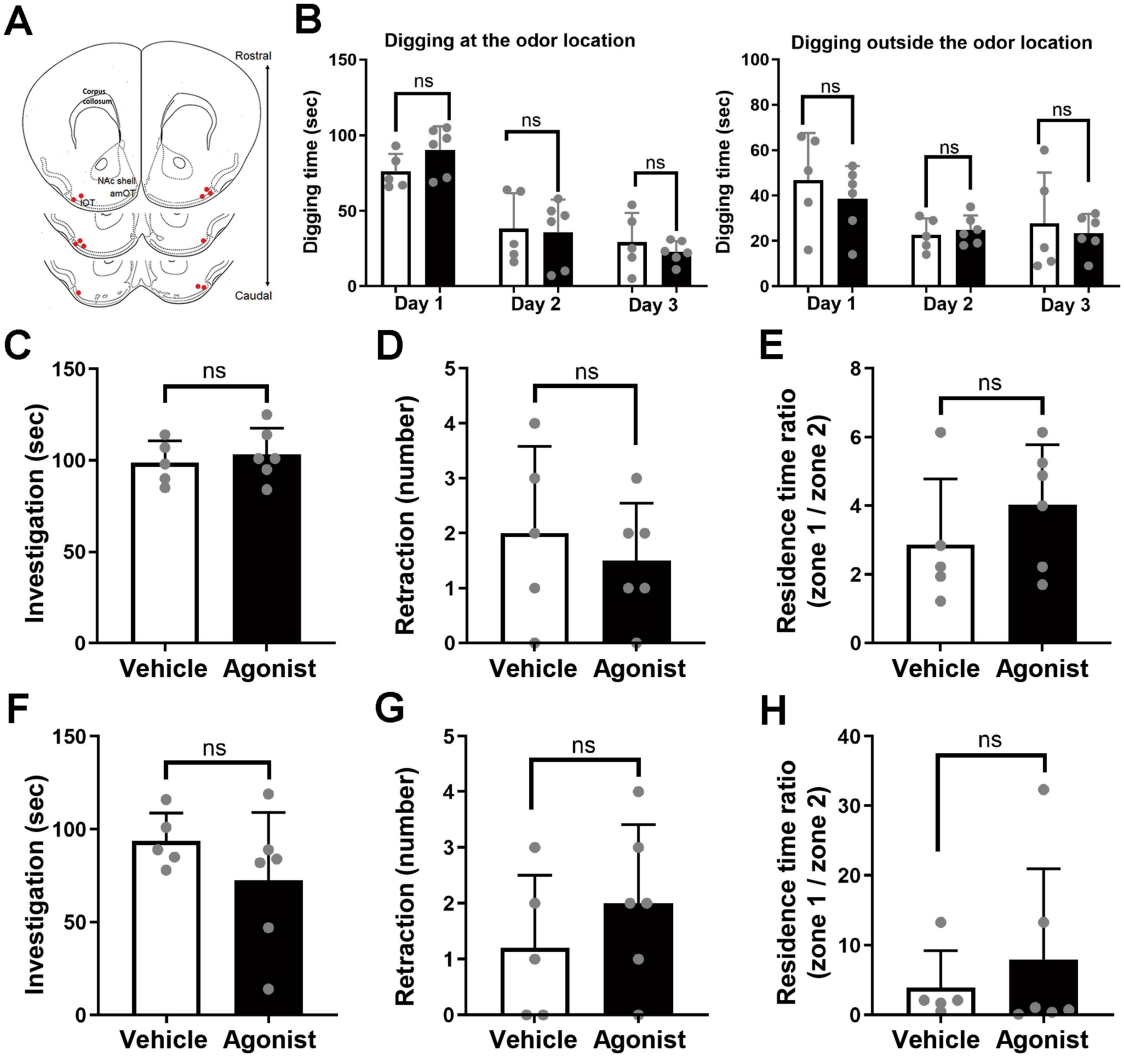
Odor-attractive behavior is not significantly influenced by the local injection of an MC4R agonist into the lOT. **(A)** Schematic of cannula placement (red dots; *n* = 6 agonist-injected mice). **(B)** Odor-guided behavior of mice during test days 1–3. (Left) Digging behavior at the odor location. (Right) Digging behavior outside the odor location. **(C–E)** Odor-guided behavior of food-restricted mice under the bedding-free condition. **(C)** Odor-investigating behavior. **(D)** Odor-retraction behavior. **(E)** Ratio of residence time on the odor side (zone 1) versus the opposite side (zone 2). **(F–H)** Odor-guided behavior of *ad libitum*-fed mice under the bedding-free condition. **(F)** Odor-investigating behavior. **(G)** Odor-retraction behavior. **(H)** Ratio of residence time on the odor side (zone 1) versus the opposite side (zone 2). White columns, vehicle-injected mice; black columns, agonist-injected mice. Average ± standard deviation, each dot represents one mouse (*n* = 5 mice for vehicle-injected group; *n* = 6 for agonist-injected group). ns, not significant (*t*-test).

**Figure 3:**
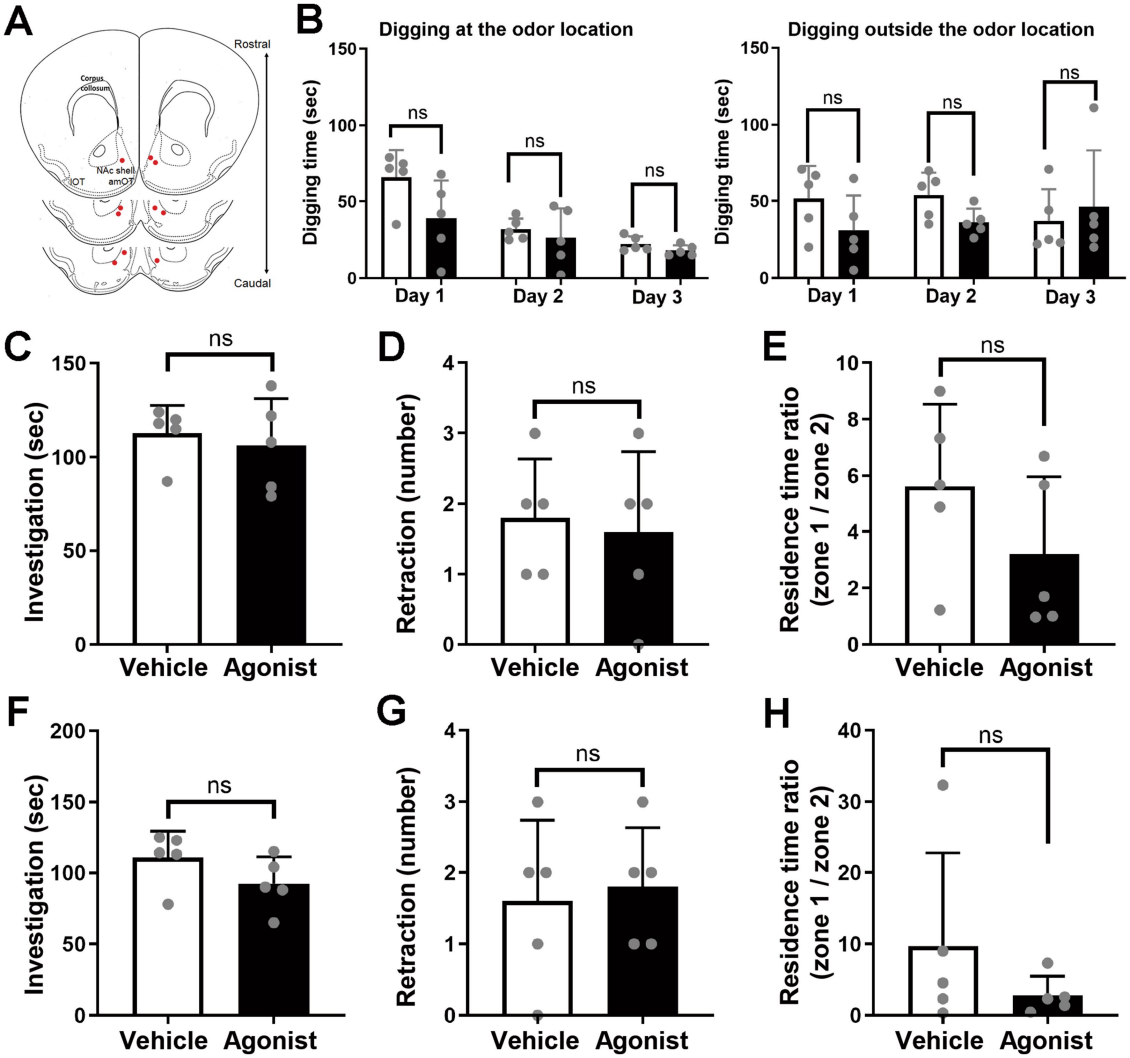
Odor-attractive behavior is unaffected by local injection of an MC4R agonist into the NAc shell. **(A)** Schematic of cannula placement (red dots; *n* = 5 agonist-injected mice). **(B)** Odor-guided behavior of mice during test days 1–3. (Left) Digging behavior at the odor location. (Right) Digging behavior outside the odor location. **(C–E)** Odor-guided behavior of food-restricted mice under the bedding-free condition. **(C)** Odor-investigating behavior. **(D)** Odor-retraction behavior. **(E)** Ratio of residence time on the odor side (zone 1) versus the opposite side (zone 2). **(F–H)** Odor-guided behavior of *ad libitum*-fed mice under the bedding-free condition. **(F)** Odor-investigating behavior. **(G)** Odor-retraction behavior. **(H)** Ratio of residence time on the odor side (zone 1) versus the opposite side (zone 2). White columns, vehicle-injected mice; black columns, agonist-injected mice. Average ± standard deviation, each dot represents one mouse (*n* = 5 mice for both groups). ns, not significant (*t*-test).

To determine whether the effect of the agonist on odor-guided behavior was specific to its injection into the amOT, mice were implanted with drug cannulas in the bilateral lOT or NAc shell and subjected to behavioral analysis. Cannula placement was confirmed histologically following completion of the experiments (Figures 2A, 3A).

Injection of the agonist into the lOT did not significantly alter attraction to the cue odor. Across test days 1–3, mice extensively dug at the odor location, and their digging behavior outside the odor location was comparable to that of vehicle-injected controls (digging at the odor location: *F*(1, 9) = 0.046, *p* = 0.84 for injection; *F*(2, 18) = 1.67, *p* = 0.22 for interaction, two-way ANOVA; *p* = 0.13, 0.85, and 0.46 for days 1, 2, and 3, respectively, *t*-test; Figure 2B, left panel) (digging outside the odor location: *F*(1, 9) = 0.30, *p* = 0.60 for injection; *F*(2, 18) = 1.67, *p* = 0.22 for interaction; *p* = 0.46, 0.63, and 0.66 for days 1, 2, and 3, respectively; Figure 2B, right panel). Under bedding-free conditions (day 4), food-restricted mice injected in the lOT showed robust odor investigation (*p* = 0.59; Figure 2C), no significant retraction behavior (*p* = 0.54; Figure 2D), and no differences in time spent on the odor side versus the opposite side (*p* = 0.32; Figure 2E). Similar results were observed under well-fed conditions after ~24 h of *ad libitum* feeding (day 5): agonist-injected mice continued to investigate the odor (*p* = 0.26; Figure 2F), did not exhibit significant retraction (*p* = 0.36; Figure 2G), and showed no shift in side preference (*p* = 0.53; Figure 2H) compared to vehicle-injected mice.

Likewise, injection of the agonist into the NAc shell did not affect odor-attractive digging behavior (digging at the odor location: *F*(1, 8) = 2.65, *p* = 0.14 for injection; *F*(2, 16) = 3.12, *p* = 0.072 for interaction; *p* = 0.083, 0.57, and 0.14 for days 1, 2, and 3, respectively; Figure 3B, left panel) (digging outside the odor location: *F*(1, 8) = 1.13, p = 0.32 for injection; *F*(2, 16) = 1.48, p = 0.26 for interaction; *p* = 0.18, 0.054, and 0.63 for days 1, 2, and 3, respectively; Figure 3B, right panel). Under bedding-free conditions, agonist injection into the NAc shell did not significantly affect odor investigation (*p* = 0.62, day 4; Figure 3C; *p* = 0.17, day 5; Figure 3F), retraction behavior (*p* = 0.76, day 4; Figure 3D; *p* = 0.76, day 5; Figure 3G), or side preference (*p* = 0.22, day 4; Figure 3E; *p* = 0.28, day 5; Figure 3H).

These findings indicate that the behavioral effects of MC4R agonist on odor-attractive and -aversive responses are specific to its injection into the amOT.

### Local injection of an MC4R antagonist into the amOT converts unassociated odor valence from neutral to attraction

To examine whether physiological MC4R signaling in the amOT contributes to odor-guided behaviors, mice received injections of the MC4R peptide antagonist, HS014, in the amOT. The mice’s behaviors to a neural odor without association learning was examined. The same odor (eugenol) was presented to food-restricted mice without a sugar reward for 4 days. Then, the behavior of the mice to the odor presentation was examined following MC4R antagonist or vehicle injection into the amOT (Figure 4). Cannula placement was confirmed histologically following completion of the experiments (Figure 4A).

Digging times at the odor location in vehicle-injected mice on days 1–3 were short compared to those observed in vehicle-injected mice that had undergone odor-reward association learning (Figure 1). By contrast, antagonist-injected mice showed significantly increased digging times at the odor location. While digging time on day 1 was comparable between groups, it significantly increased in antagonist-injected mice on days 2 and 3 (*F*(1, 9) = 20.68, *p* = 0.001 for injection; *F*(2, 18) = 0.50, *p* = 0.62 for interaction, two-way ANOVA; test day 1, *p* = 0.17; test day 2, *p* = 0.003; test day 3, *p* = 0.024; *t*-test; Figure 4B, left panel). Digging outside the odor location remained low in both groups across days 1–3 with no significant differences in the comparison within each day (test day 1, *p* = 0.27; test day 2, *p* = 0.068; test day 3, *p* = 0.063; *t*-test; Figure 4B, right panel), but showed slight decrease in the antagonist-injected group in the comparison across 3 days (*F*(1, 9) = 5.48, *p* = 0.044 for injection; *F*(2, 18) = 0.32, *p* = 0.73 for interaction, two-way ANOVA), presumably reflecting their attraction to the odor location.

Simple behavioral tests without bedding were conducted on day 4 under food-restricted conditions and on day 5 following ~24 h of *ad libitum* feeding, during which body weights returned to pre-restriction levels. The body weights of antagonist-injected mice just before food restriction, during restriction (test day 4), and after *ad libitum* feeding (test day 5) were 25.5 ± 0.7 g, 21.7 ± 0.6 g (85.1 ± 1.3% of that just before food restriction), and 26.7 ± 1.4 g (104.7 ± 3.6%; *n* = 6), respectively. For vehicle-injected mice, the corresponding values were 26.1 ± 1.2 g, 22.4 ± 0.9 g (85.7 ± 2.3%), and 27.4 ± 1.5 g (105.1 ± 2.9%; *n* = 5).

Odor-investigation time increased in antagonist-injected mice on both food-restricted day 4 (*p* = 0.036, *t*-test; Figure 4C) and well-fed day 5 (*p* = 0.046; Figure 4F). Odor-retraction behavior did not significantly differ on either day (day 4, *p* = 0.69; Figure 4D; day 5, *p* = 0.49; Figure 4G). The ratio of time spent in the odor-side zone (zone 1) versus the opposite side (zone 2) was also not significantly different (day 4, *p* = 0.57; Figure 4E; day 5, *p* = 0.21; Figure 4H), possibly due to reduced motivation to investigate a neutral, unassociated odor.

These findings indicate that local MC4R antagonist injection into the amOT converts the valence of an unassociated odor from neutral to attractive.

### Injection of an MC4R agonist into the NAc shell induces yawning and stretching behaviors following an elevation of brain temperature

During behavioral analysis of mice that received MC4R agonist injection into the NAc shell, we occasionally observed yawning and stretching behaviors. These included wide mouth opening with closed eyes (yawning) and limb extension with backward trunk bending (stretching), consistent with established definitions (Gupta and Mittal, 2013; Krestel et al., 2018) (Supplementary Video 5). These behaviors were not observed in mice that received MC4R agonist injection into the amOT or lOT.

**Figure 5:**
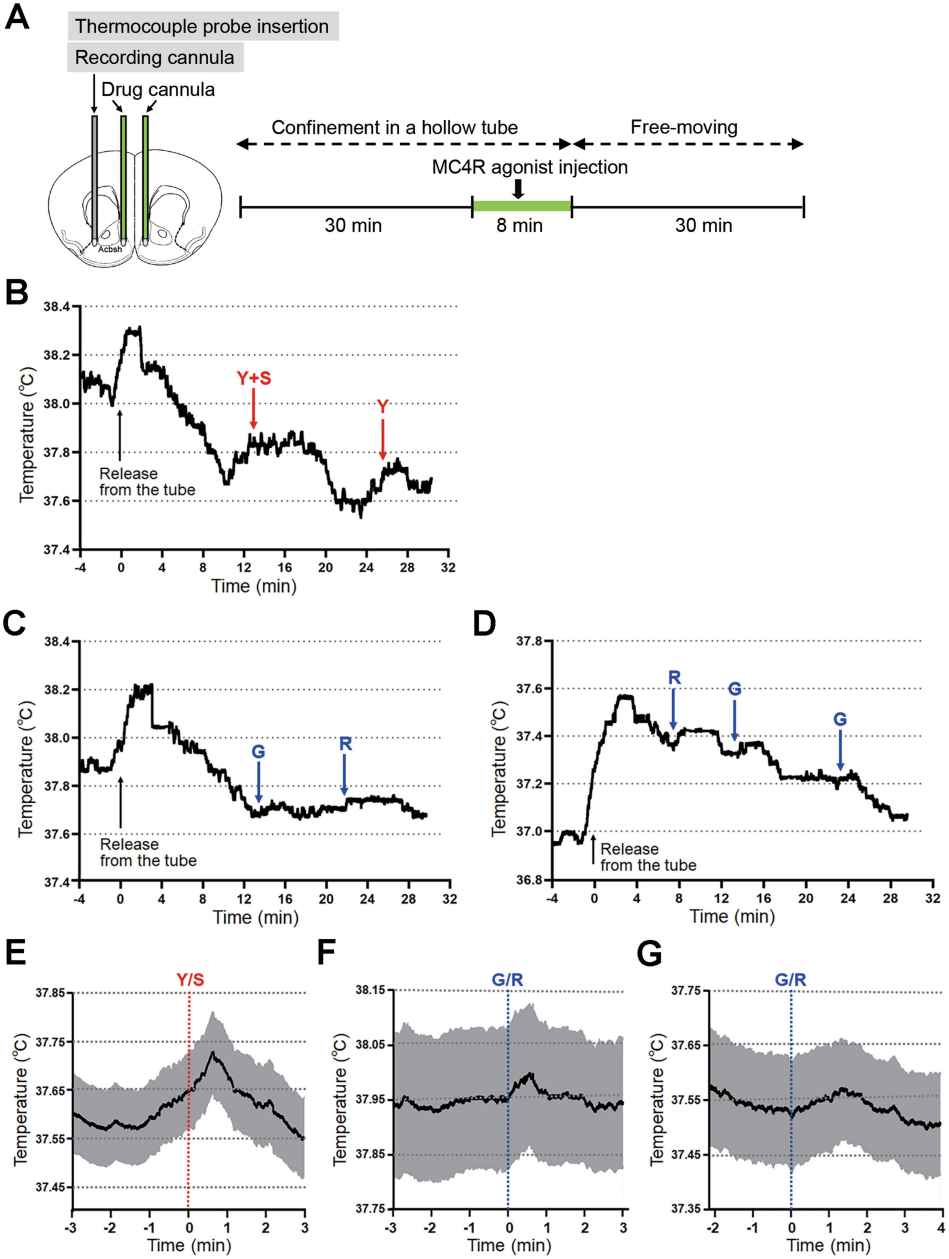
Promotion of yawning and stretching behavior by local injection of an MC4R agonist into the NAc shell. **(A)** Protocol for the behavioral analysis and brain temperature recordings following MC4R agonist injection. *Ad libitum*-fed mice were confined in a hollow tube before and during MC4R agonist injection. After the agonist injection, the mice were released from the tube and allowed to move freely in a cage. **(B)** A representative shift in brain temperature before, during, and after the yawning/stretching behaviors, following MC4R agonist injection in the NAc shell. Time 0 indicates release time from the tube. Y, yawning; S, stretching. **(C)** A representative shift in brain temperature before, during, and after the grooming/running behaviors, following vehicle injection in the NAc shell. Time 0 indicates release time from the tube. G, grooming; R, running. **(D)** A representative shift in brain temperature before, during, and after the grooming/running behaviors, following MC4R agonist injection in the amOT. Time 0 indicates release time from the tube. **(E)** Average shift in brain temperature before, during, and after the yawning/stretching behaviors. Twelve events of yawning/stretching behaviors were extracted from five mice. The brain temperature was aligned by setting the starting time of the yawning/stretching behaviors (Y/S) as time 0. **(F)** Average shift in brain temperature of mice that received vehicle injection into the NAc shell. Nine events of grooming/running behavior were extracted from five mice. The brain temperature before, during, and after the behavior was aligned by setting the starting time of the grooming/running behaviors (G/R) as time 0. **(G)** Average shift in brain temperature of mice that received MC4R agonist injection into the amOT. Ten events of grooming/running behavior were extracted from four mice. The brain temperature was aligned by setting the starting time of the grooming/running behaviors (G/R) as time 0. In **E–G**, average (black line) and ± standard error of mean (shaded area) are indicated.

Yawning and stretching have been linked to various physiological mechanisms, and many pharmacological agents induce these behaviors (Argiolas and Melis, 1998; Gupta and Mittal, 2013; Krestel et al., 2018). A thermoregulatory hypothesis suggests that yawning/stretching may be triggered by elevated brain temperature as a cooling mechanism (Gallup and Eldakar, 2012; Shoup-Knox et al., 2010). To test this, we implanted a guide cannula for a thermocouple probe unilaterally in the lateral NAc, along with bilateral drug cannulas in the NAc shell. A thermocouple probe was inserted through the guide cannula, and the mouse was held in a hollow tube before and during MC4R agonist injection to ensure immobilization for proper agonist injection, and released from the tube after injection to allow free movement in a test cage (Figure 5A).

Behavior and brain temperature were monitored before (30 min), during (8 min), and after (30 min) MC4R agonist injection. In five mice analyzed, yawning/stretching occurred occasionally after injection, with each event lasting several seconds. Figure 5B shows a representative brain temperature trace. Immediately after releasing from the tube (time 0), brain temperature elevated along with the active movement (running) in the cage. Then, while the temperature gradually declined, it began increase again, and yawning/stretching occurred afterward.

Across five mice, 12 yawning/stretching events were recorded: six yawning alone, two stretching alone, and four combined events (Table 1). The latency from injection to behavior onset ranged from 8 min 6 s to 28 min 20 s (median: 19 min 57 s). Importantly, all events were preceded by brain temperature elevation, with latency from temperature increase to behavior onset ranging from 10 s to 2 min 35 s (median: 49 s). After the behavior, brain temperature peaked and then declined. Time from behavior onset to temperature peak ranged from 4 s to 1 min 25 s (median: 35 s), and time to return to baseline ranged from 20 s to 3 min 0 s (median: 2 min 0 s). The magnitude of temperature elevation ranged from 0.05 °C to 0.28 °C (median: 0.16 °C).

**Table 1:** Yawning/stretching events and brain temperature.

Mouse	Behavior	Time from:	End of agonist inj.	Start of temp. Rise	Onset of Y/S	Peak of temp.	Temp. increase
		To:	Onset of Y/S	Onset of Y/S	Peak of temp.	Return of temp.	
#1	S		8:06	0:10	0:04	1:57	0.11
	Y		19:28	0:38	0:34	0:20	0.28
	Y		20:26	0:12	0:18	0:34	0.14
#2	Y + S		23:09	1:44	1:16	3:00	0.10
	Y		24:11	0:10	0:15	1:52	0.05
#3	Y		14:55	0:15	0:25	0:46	0.09
	Y + S		15:56	1:18	0:52	2:18	0.17
	S		25:53	0:25	0:35	2:10	0.19
#4	Y + S		8:37	1:45	0:05	0:40	0.15
	Y		13:16	1:00	0:40	2:23	0.18
#5	Y + S		22:20	2:14	0:45	3:00	0.20
	Y		28:20	2:35	1:25	2:02	0.23
Median			19:57	0:49	0:35	2:00	0.16

Twelve events of yawning/stretching from 5 mice are indicated. Y, yawning; S, stretching.

Time from one point to another point is indicated as [min:sec].

“End of agonist inj.” indicates the time point when MC4R agonist injection into bilateral NAc shell was completed.

“Start of temp. Rise” indicates the time point when the brain temperature started to rise.

“Onset of Y/S” indicates the time point when the yawning/stretching event started.

“Peak of temp.” indicates the time point when the brain temperature increased to a peak.

“Return of temp.” indicates the time point when the brain temperature went down to the value at the start of temperature rise.

“Temp. increase” indicates the increased value of the temperature as [°C] from the start of rise to the peak.

Figure 5E shows the average brain temperature trajectory aligned to the onset of the 12 yawning/stretching events (time 0). Average latency from the start of temperature increase to behavior onset was 1 min 20 s. Temperature peaked at 37 s post-onset and returned to baseline by 2 min 3 s. The average temperature elevation was 0.16 ± 0.05 °C (average ± standard deviation).

By contrast, vehicle injection into the NAc shell did not induce yawning/stretching or significant brain temperature elevation. A representative brain temperature trace showed immediate elevation after releasing from the tube and gradual decline (Figure 5C). Relatively small brain temperature elevation was occasionally observed, during vigorous behaviors such as grooming or running. In five mice analyzed, 9 events of brain temperature elevation were observed (except for the steep elevation following the release from the tube), but the elevations were only observed during vigorous behaviors of grooming or running, and the elevations were small in magnitude ranging from 0.034 °C to 0.080 °C (0.052 ± 0.014 °C; average ± standard deviation) (Figure 5F; the average brain temperature trajectory aligned to the onset of the 9 grooming/running events). Similarly, MC4R agonist injection into the amOT did not elicit yawning/stretching or significant temperature changes. A representative brain temperature trace showed immediate elevation after releasing from the tube, gradual decline and occasional elevation during grooming or running (Figure 5D). In four mice analyzed, 10 events of brain temperature elevation were observed only during vigorous behaviors of grooming or running, and the magnitude of temperature elevation ranged from 0.039 °C to 0.070 °C (0.052 ± 0.010 °C; average ± standard deviation) (Figure 5G; the average brain temperature trajectory aligned to the onset of the 10 grooming/running events).

These findings indicate that yawning/stretching induced by MC4R agonist is specific to injection into the NAc shell and is temporally linked to preceding brain temperature elevation and subsequent decline.

## Discussion

We showed that local injection of an MC4R agonist into the amOT, but not into the lOT or NAc shell, converted odor valence from attraction to aversion. Conversely, local injection of an MC4R antagonist into the amOT shifted odor valence from neutral to attraction. By contrast, local injection of an MC4R agonist into the NAc, but not the amOT, induced yawning and stretching behaviors, which were preceded by elevated brain temperature. These findings highlight the differential roles of MC4R signaling within the ventral striatum and suggest their combined contribution to behavioral regulation under feeding conditions.

### Functions of MC4R signaling in the amOT in odor-guided motivation behaviors

While the relationship between metabolic status and olfaction has been examined for various neuromodulatory and nutrient molecules (Julliard et al., 2017; Palouzier-Paulignan et al., 2012), knowledge of the melanocortin–MC4R system in olfactory processing remains limited. MC4R knockout mice, widely used as a genetic model of obesity, exhibit multiple deficits in olfactory behaviors (Tucker et al., 2012). In addition, MC4R signaling in the parabrachial nucleus of the brainstem has been implicated in the suppression of odor-guided food-seeking behavior (Shionoya et al., 2023). However, the role of MC4R signaling within central olfactory neural circuits has yet to be fully elucidated.

In this study, we demonstrated that MC4R signaling in the amOT plays a critical role in regulating odor valence and odor-guided behavior. Mice that underwent odor-sugar reward conditioning exhibited extensive digging behavior directly above the cue odor, likely reflecting both odor-approach and food-seeking components. Under bedding-free conditions, odor investigation also included these behavioral elements, which we collectively referred to as odor-attractive behavior. In contrast, mice injected with MC4R agonist in the amOT displayed digging behavior away from the odor source and retraction from the odor under bedding-free conditions. This displaced digging may represent sheltering from a perceived threat (Fucich and Morilak, 2018; Harper and Batzli, 1996), or odor-induced anxiety which can potentiate aberrant behaviors such as marble burying (Himanshu et al., 2020). During odor retraction, the mice initially oriented themselves toward the odor and occasionally approached it, but subsequently withdrew. This behavioral sequence implies an initial vigilance toward the odor as a potential threat (Zhao et al., 2024), followed by a fear-driven escape response that may be amplified by odor-induced anxiety. We collectively refer to these motivational states observed in MC4R agonist-injected mice as odor-aversive behavior.

Consistent with the broader function of MC4R in suppressing appetitive and consummatory behaviors, activation of MC4R in the amOT shifted the valence of food-associated odors from attraction to aversion. Conversely, local injection of an MC4R antagonist converted the valence of an unassociated odor from neutral to attractive. Most of these effects were evident as early as 30 min after drug injection (Figures 1, 4), aligning with everyday experience in which food odors become less appealing when we are satiated. Moreover, the behavioral effects appeared to be potentiated over the 3-day test period, implicating the OT in odor valence learning and behavioral adaptation (Gadziola et al., 2020; Gadziola et al., 2015; Martiros et al., 2022; Murata et al., 2015; Sha et al., 2023). These immediate and progressive effects of MC4R signaling in the amOT parallel those observed for OxR1 signaling in the same region (Ahasan et al., 2024), suggesting that the amOT may serve as a hub for integrating odor information with metabolic signals to adaptively regulate odor valence.

In addition to the appetite-promoting OxR1 signaling in the amOT (Ahasan et al., 2024), we demonstrated the contribution of appetite-suppressing MC4R signaling in the amOT to odor valence control. These findings support the notion that the amOT is a specialized brain region that regulates odor valence bidirectionally in a metabolic status-dependent manner by integrating both appetite-promoting and -suppressing signals. Notably, the amOT also expresses high levels of appetite-promoting ghrelin receptors and appetite-suppressing arginine-vasopressin receptors (Nogi et al., 2020). Further investigation of these molecular pathways may enhance our understanding of the amOT as an integrative hub for odor–metabolism interactions and reveal potential mechanisms of cross-talk among diverse metabolic signals in this region.

Although knowledge of MC4R signaling in olfactory brain regions remains limited, numerous studies have characterized its role in the NAc. Injection of melanocortin receptor agonists into the NAc has been shown to reduce both appetitive and consummatory responses to food (Eliason et al., 2022), and MC4R agonist and antagonist injections, respectively decrease and increase food intake (Lerma-Cabrera et al., 2012). Furthermore, melanocortin–MC4R signaling in the NAc enhances non-appetitive aversive behaviors, such as anhedonia and avoidance of aversive stimuli (Klawonn et al., 2018; Lim et al., 2012). Intriguingly, in our study, MC4R agonist injection into the NAc did not affect odor-attractive behavior. This dissociation suggests that odor valence and feeding motivation can be differentially regulated by MC4R signaling in distinct regions of the ventral striatum. We showed that the effects of MC4R agonist or antagonist injection in the amOT on odor valence were not significantly altered by the hunger-satiety state (Figures 1, 4; test days 4 and 5). However, impact of MC4R signaling in the amOT on appetitive behaviors remains unexplored. Self-administration studies have demonstrated a stronger reward effect of cocaine in the anteromedial OT region compared to the NAc shell (Ikemoto, 2003). Future investigations may uncover a role for the amOT in MC4R-mediated regulation of appetite.

### Functions of MC4R signaling in the NAc in yawning/stretching behaviors

Yawning is characterized by a sequence of respiratory phases involving a long, deep inhalation followed by rapid expiration, typically accompanied by wide mouth opening and closed eyes (Gupta and Mittal, 2013; Krestel et al., 2018). It is frequently paired with stretching of the limbs and trunk. In the present study, we observed typical yawning and stretching behaviors following MC4R agonist injection into the NAc shell. Although multiple functions have been proposed for yawning, ranging from recovery from cerebral hypoxemia and hypercapnia, stimulation of arousal, and social communication to brain cooling, these are not mutually exclusive (Gupta and Mittal, 2013; Krestel et al., 2018). In rats, yawning, and stretching occur during brain temperature elevation, followed by a return to baseline (Shoup-Knox et al., 2010). Mechanistically, wide mouth opening during yawning increases blood flow in the neck, head, and face, while deep inhalation enhances cerebrospinal fluid movement and venous return via the internal jugular vein, all of which may contribute to brain cooling (Gallup and Eldakar, 2012; Schroth and Klose, 1992; Zajonc, 1985). In humans, yawning frequency increased during the rising phase of body temperature induced by lipopolysaccharide injection, although brain temperature was not directly measured (Marraffa et al., 2017). Consistent with these findings, we observed that MC4R agonist injection into the NAc shell led to transient brain temperature elevation, followed by yawning/stretching behaviors and subsequent temperature decline (Figure 5), supporting a thermoregulatory role for these behaviors.

To our knowledge, yawning/stretching induced by activation of the melanocortin system has not been previously reported. However, various neurotransmitters and hormones, including excitatory amino acids, acetylcholine, dopamine, and adrenocorticotropic hormone, stimulate yawning (Argiolas and Melis, 1998). In humans, drug-induced yawning is most frequently associated with apomorphine, a classical dopamine receptor agonist (Mohandoss and Thavarajah, 2023), and similar effects have been observed in rodents (Mogilnicka and Klimek, 1977). Notably, interactions between melanocortin and dopamine signaling in the NAc have been documented. For example, stress-induced anhedonia requires MC4R-mediated synaptic adaptations in dopamine receptor 1-expressing (D1) neurons in the NAc (Lim et al., 2012), and the reversal of aversive stimulus valence in MC4R-deficient mice is dopamine-dependent, restored by re-expression of MC4R in striatal D1 neurons (Klawonn et al., 2018). Thus, the yawning observed following MC4R agonist injection may be mediated by dopaminergic signaling in the NAc. Although the precise location of the yawning motor center has not been identified, it is suggested to reside within the brainstem spanning from the midbrain to the medulla oblongata (Krestel et al., 2018). The paraventricular nucleus of the hypothalamus (PVH) has been proposed to regulate this motor center (Krestel et al., 2018). Given that yawning and stretching behaviors and the elevation of brain temperature occurred intermittently at various times after the agonist injection, it is likely that multiple brain regions and downstream signaling pathways of MC4R activation in the NAc contribute to these phenomena. Activation of the dopaminergic pathway via reciprocal connections between the NAc and the ventral tegmental area (Ikemoto, 2007) may, in turn, stimulate the PVH-yawning motor circuitry (Argiolas and Melis, 1998). The preoptic area of the hypothalamus is the central brain region for thermoregulation (Morrison and Nakamura, 2019), and the link between hypothalamic dopamine signaling and thermoregulation has been reported (Folgueira et al., 2019). Further investigation into the interaction between melanocortin and dopamine pathways will be essential for understanding the mechanisms underlying MC4R agonist-induced yawning/stretching and brain thermoregulation.

### Role of MC4R signaling in behaviors under satiation

The melanocortin–MC4R system is a central regulator of feeding behavior and energy expenditure, with its activation reflecting a satiated and energized physiological state (Baldini and Phelan, 2019; Krashes et al., 2016). In terms of odor valence, food odors typically elicit appetite when we are hungry, but the same odors may lose their appeal, or even evoke nausea, when we are satiated. The present findings suggest that this feeding-related shift in odor valence may be mediated, at least in part, by MC4R signaling in the amOT. By integrating MC4R-mediated, OxR1-mediated (Ahasan et al., 2024), and other feeding-related signals, the amOT may adaptively regulate odor valence in accordance with hunger–satiety and energy status.

Satiation following food intake induces subjective sleepiness (Orr et al., 1997), and sleepiness is a well-established trigger for yawning (Gupta and Mittal, 2013; Krestel et al., 2018). Notably, rats consuming high-fat chow exhibited increased sensitivity to apomorphine-induced yawning (Baladi et al., 2012). These observations support the idea that satiety signals, such as those mediated by the melanocortin–MC4R system, may contribute to the induction of yawning. Although the biological significance of postprandial sleepiness and yawning remains unclear, MC4R signaling in the ventral striatum may play a role in coordinating postprandial motivational and behavioral states, including reduced attraction to food odors and the emergence of sleepiness-associated behaviors such as yawning.

### Future perspectives

The link between metabolic and energy status and affective or motivational states is highly complex, involving diverse molecules, cell types, brain regions, and peripheral organs. To elucidate the underlying mechanisms, it is essential to dissect the relevant neural circuits and characterize their individual properties. In the striatum, neurons are broadly categorized into D1 and type 2-expressing (D2) cells. These cell types play complementary roles in the NAc (Hikida et al., 2016), and similar complementary functions have been suggested in the OT in the context of behavioral regulation (Gadziola et al., 2020; Martiros et al., 2022; Murata et al., 2019). Understanding how the MC4R signaling pathway interacts with D1 and/or D2 cell pathways in the ventral striatum is an important direction for future research. In the NAc, MC4R-dependent anhedonia and aversive responses are mediated via D1 cells (Klawonn et al., 2018; Lim et al., 2012), and whether similar mechanisms operate in the OT remains to be determined.

Concerning the possible gender effect of MC4R signaling, only male mice were used in the current analysis. A single nucleotide polymorphism that is thought to influence MC4R expression in humans has been shown to have a stronger effect on eating behavior in females (Horstmann et al., 2013). In rodents, MC4R expression level showed sex differences in certain brain regions (Gelez et al., 2010), and MC4R loss-of-function is more often associated with stress reactivity in females (Chaffin et al., 2019). Thus, potential sex differences in the effects of MC4R signaling on odor valence and yawning/stretching warrant further investigation.

Eating disorders and overeating have been associated with dysfunction of the brain’s reward system (Berridge et al., 2010; Stuber et al., 2025). The melanocortin system has emerged as a promising therapeutic target for metabolic disorders (Sweeney et al., 2023). In particular, hedonic responses to food odors and dopaminergic activity are disrupted in eating disorders, particularly in individuals with anorexia nervosa (Jiang et al., 2019). Modulating melanocortin–MC4R signaling in the ventral striatum may help restore normal eating behavior by influencing food odor hedonics and feeding motivation. Continued investigation into the neural circuits and functional roles of the melanocortin–MC4R system in the ventral striatum will advance our understanding of feeding-related motivated behaviors and support the development of targeted interventions for eating disorders.

## Data Availability

The original contributions presented in the study are included in the article/Supplementary material, further inquiries can be directed to the corresponding author.

## Supplementary material

SUPPLEMENTARY VIDEO 1 Digging behavior of a vehicle-injected mouse. The vehicle-injected mouse is extensively digging at the location of the odor (under the bedding on the left side) which was associated with sugar reward.

SUPPLEMENTARY VIDEO 2 Digging behavior of a MC4R agonist-injected mouse. The MC4R agonist-injected mouse is extensively digging outside the odor location, mostly on the side opposite the odor location (right side).

SUPPLEMENTARY VIDEO 3 Odor-investigating behavior of a vehicle-injected mouse. The vehicle-injected mouse is extensively investigating the dish scented with the cue odor.

SUPPLEMENTARY VIDEO 4 Odor-retraction behavior of a MC4R agonist-injected mouse. The MC4R agonist-injected mouse first approaches the scented dish with its body in longitudinal extension, and then rapidly retracts its head and body from the dish. The mouse is mostly staying on the side opposite the odor.

SUPPLEMENTARY VIDEO 5 Yawning and stretching behavior of a mouse injected with MC4R agonist in the NAc shell. The mouse injected with MC4R agonist in the NAc shell shows yawning (wide mouth opening with closed eyes) and stretching (limb extension with backward trunk bending).
