# Supplementary material for: Differential potentiation of odor aversion and yawning by melanocortin 4 receptor signaling in distinct regions of the ventral striatum
Source: Front Neurosci. 2025 Nov 4;19:1668410. doi: 10.3389/fnins.2025.1668410 (PMC12623337; doi:10.3389/fnins.2025.1668410)
Supplement: Supplementary file 6 [file Data_Sheet_1.pdf]

Supplementary Material

Differential potentiation of odor aversion and yawning by melanocortin 4 receptor signaling in distinct regions of the ventral striatum

Md Tasnim Alam, Md Monjurul Ahasan, Shogo Shimizu, Yoshihiro Murata, Mutsuo Taniguchi, Masahiro Yamaguchi*

Department of Physiology, Kochi Medical School, Kochi University, Kochi, Japan

*** Correspondence:**
Masahiro Yamaguchi
yamaguchi@kochi-u.ac.jp

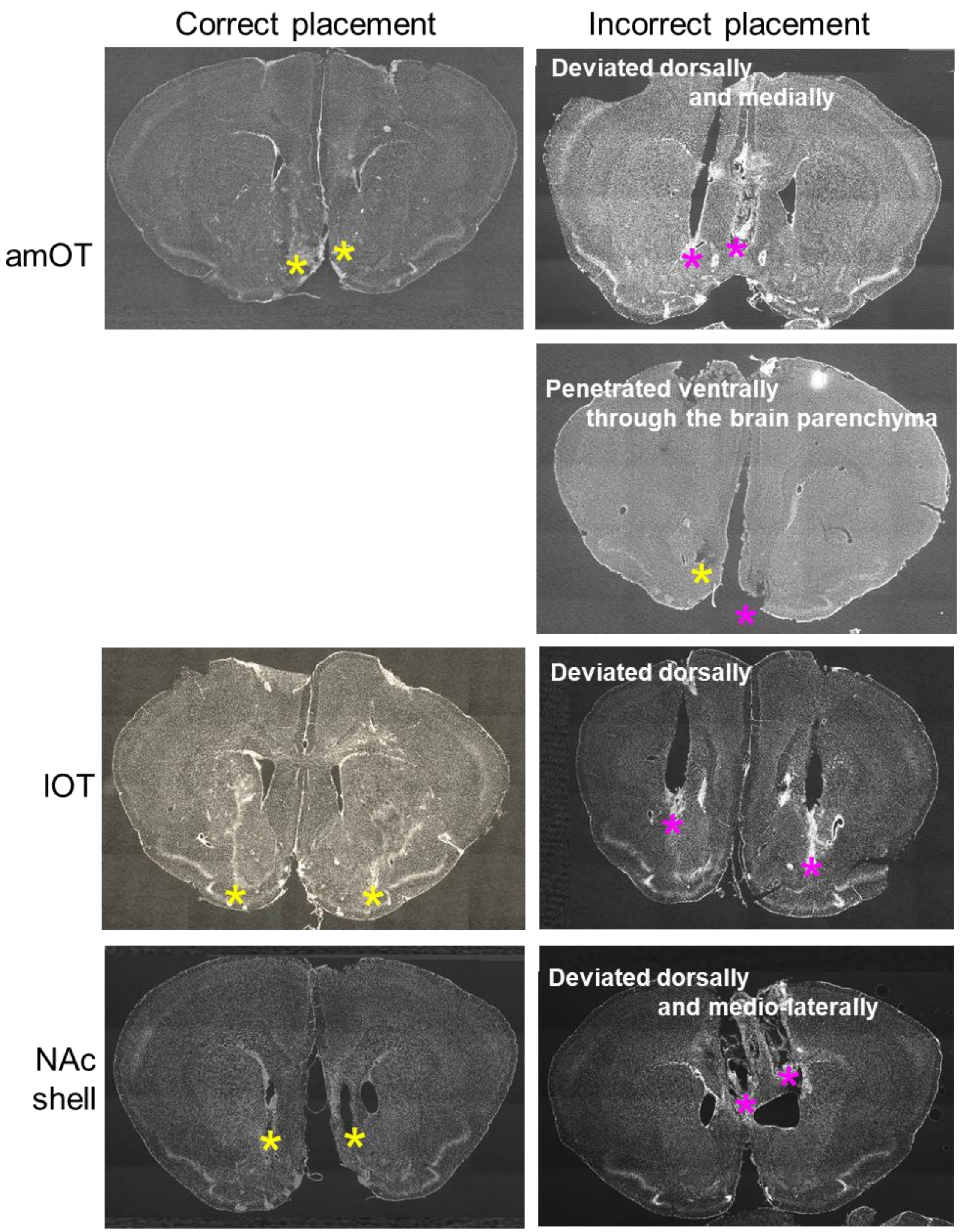

Supplementary Fig. 1. Placement of drug cannulas

Representative correct (left panels; yellow asterisks) and incorrect (right panels; pink asterisks) cannula placements in the amOT, lOT and NAc shell are indicated in coronal sections of the mouse brain stained with DAPI. Mice with incorrect cannula placement were excluded from analysis.

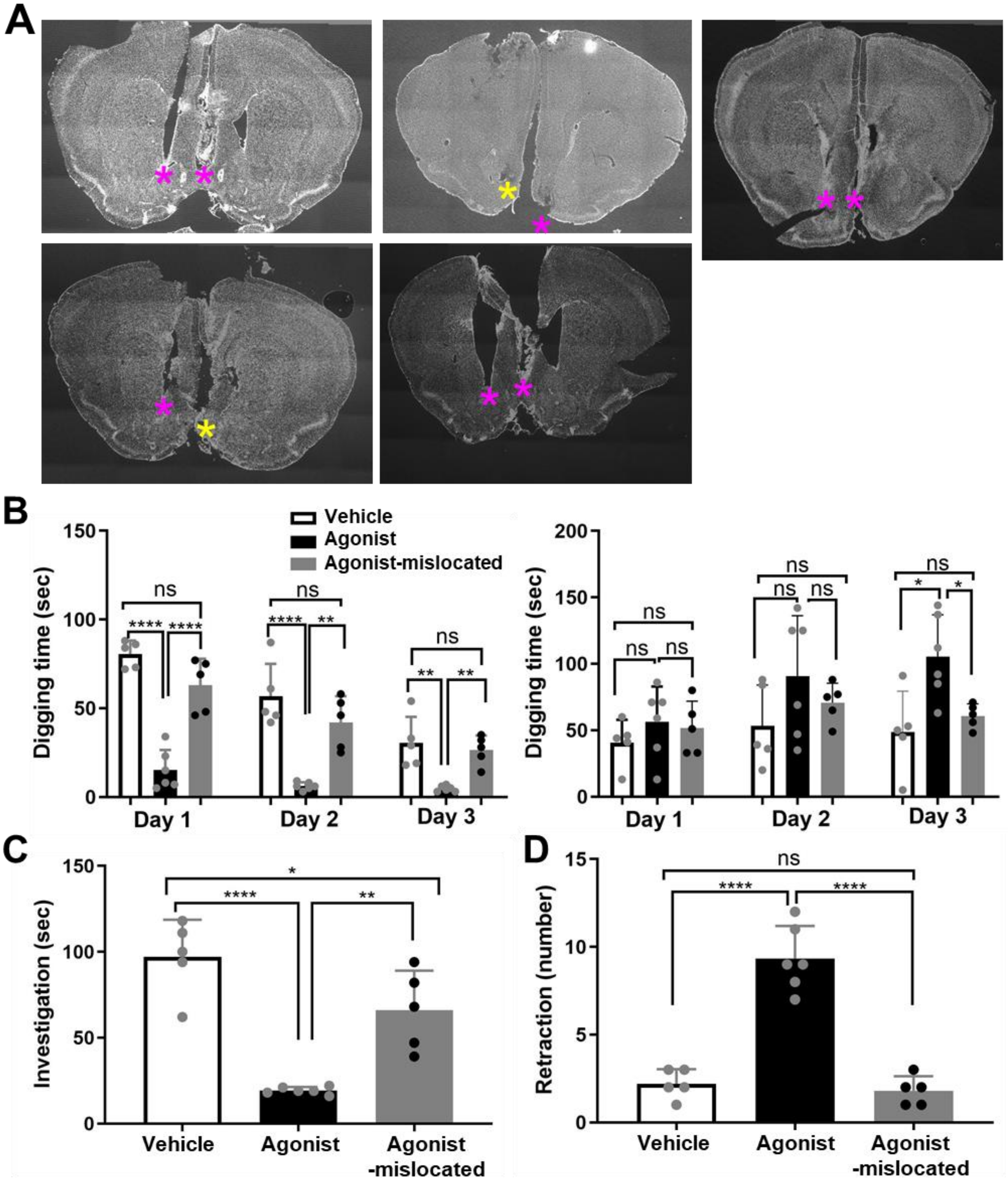

E

p values in Tukey's multiple comparions test

Compared pair	2B-left Day 1	2B-left Day 2	2B-left Day 3	2B-right Day 1	2B-right Day 2	2B-right Day 3	2C	2D
Vehicle vs. Agonist	< 0.0001	< 0.0001	0.0014	0.503	0.206	0.019	< 0.0001	< 0.0001
Vehicle vs. Agonist-mislocated	0.077	0.214	0.762	0.717	0.705	0.755	0.039	0.214
Agonist vs. Agonist-mislocated	< 0.0001	0.0016	0.0054	0.943	0.617	0.037	0.0028	< 0.0001

Supplementary Fig. 2. Behavior of mice with incorrect cannula placement for the experiment of MC4R agonist injection in the amOT

(A) Coronal sections of five mice's brain with incorrect cannula placements stained with DAPI. Correct (yellow asterisks) and incorrect (pink asterisks) cannula placements in the amOT are indicated.

(B) Odor-guided behavior of three groups of mice during test days 1–3. (Left) Digging behavior at the odor location. (Right) Digging behavior outside the odor location.

(C, D) Odor-guided behavior of three groups of food-restricted mice under the bedding-free condition. (C) Odor-investigating behavior. (D) Odor-retraction behavior.

(E) p-values in Tukey's multiple comparisons test for (B-D).

White columns, vehicle-injected mice via properly-located cannulas; black columns, agonist-injected mice via properly-located cannulas; gray columns, agonist-injected mice via mislocated cannulas. Data for vehicle- and agonist-injected mice via properly-located cannulas are same as those in Fig. 1. Average ± standard deviation, each dot represents one mouse (n = 5 mice for vehicle-injected via properly-located cannulas group; n = 6 for agonist-injected via properly-located cannulas group, n = 5 for agonist-injected via mislocated cannulas group). ns, not significant; *, $p < 0.05$; **, $p < 0.01$; ****, $p < 0.0001$ (Tukey's multiple comparisons test).
